# Participation of the Periosteum, Endosteum, and Hematogenous Marrow in the Early Osseointegration of a Titanium Implant Inserted in Contact with the Hematogenous Marrow

**DOI:** 10.3390/medicina61101841

**Published:** 2025-10-14

**Authors:** Cristian Adrian Ratiu, Cosmin Sinescu, Danut Dejeu, Ovidiu Tica, Corina Moisa, Camelia Anca Croitoru, Ioana Adela Ratiu, Virgil-Florin Duma, Adrian Todor, Viorel Miclaus, Vasile Rus

**Affiliations:** 1 Faculty of Medicine and Pharmacy, University of Oradea, 1 Decembrie Ave., No. 10, 410073 Oradea, Romania; cristian.ratiu@didactic.uoradea.ro (C.A.R.); ddejeu@uoradea.ro (D.D.); tica.ovidiu@didactic.uoradea.ro (O.T.); cmoisa@uoradea.ro (C.M.); camelia.croitoru@didactic.uoradea.ro (C.A.C.); 2 Research Center in Dental Medicine Using Conventional and Alternative Technologies, “Victor Babes” University of Medicine and Pharmacy Timisoara, 9 Revolutiei 1989 Ave., 300070 Timisoara, Romania; minosinescu@yahoo.com; 3 3OM Optomechatronics Group, Department of Measurements and Optical Electronics, Faculty of Electronics, Telecommunications, and Information Technology, Polytechnic University of Timisoara, 2 Vasile Parvan Ave., 300223 Timisoara, Romania; 4 Faculty of Medicine and Pharmacy, University of Medicine and Pharmacy “Iuliu Hatieganu” Cluj-Napoca, 8 Victor Babeș Ave., 400347 Cluj-Napoca, Romania; atodor81@gmail.com; 5 Faculty of Veterinary Medicine, University of Agricultural Sciences and Veterinary Medicine in Cluj-Napoca, Calea Mănăştur 3-5, 400372 Cluj-Napoca, Romania; vmiclaus@usamvcluj.ro (V.M.); vasile.rus@usamvcluj.ro (V.R.)

**Keywords:** periosteum, endosteum, hematogenous marrow, titanium implant, bone proliferation, osseointegration, animal study, histological analysis

## Abstract

*Background and Objectives*: Implant osseointegration has been widely studied over the past few decades, particularly focusing on surface modifications that aim to improve integration. However, the literature includes few studies regarding the role of the endosteum in early osteointegration. Therefore, the aim of the present work is to approach the technique of implant insertion into bones with marrow, with an emphasis on the use of implants that are long enough to achieve marrow penetration. *Materials and Methods*: Titanium implants were inserted into the femurs of eight eleven-month-old rabbits. Fourteen days later, the animals were euthanized in accordance with the current legal and ethical guidelines. The histological processes that occur at the bone–implant interface were investigated. Histological sections of the bone–implant interface were colored using the Trichrome’s Goldner method, and were further analyzed and photographed using an Olympus microscope. *Results*: The histological analysis showed that during the initial osteointegration phases, the newly formed bone originated from the endosteal–medular zone. Periostal proliferation was observed only during the early stages. The bone that proliferated on the implant’s endosteal/medullary interface exhibited a surface area approximately 6 times larger than that of the bone formed on the osteal surface. Also, its length was approximately 25% longer. *Conclusions*: The bone tissue that proliferates on the endosteal and marrow surface of the implant increases significantly the bone–implant interface, and creates the setting for a good secondary stability. The findings suggest several clinical implications, as follows: penetrating the bone marrow during the insertion of short implants allows for increasing the bone–implant interface; the flap should be carefully managed; the integrity of the periosteum must be kept, as this is a key anatomical structure in cases of deficient bone marrow (i.e., severe mandible atrophy or vertical ridge augmentations).

## 1. Introduction

Bone formation is a complex process that begins during fetal development and is continued postnatally through remodeling processes. Osteogenesis occurs through two different mechanisms, termed intramembranous ossification and endochondral ossification. The former occurs mostly in the bones of the skull, mandible, maxilla, and middle clavicle, while the latter occurs in most skeletal bones [1]. During intramembranous ossification, the direct differentiation of mesenchymal precursors into osteoblasts takes place, while in the case of endochondral ossification, a cartilaginous model is initially formed and then invaded by blood vessels. Thus, it leads to bone and medullary cavity formation. During this process, hematopoietic stem cells (HSCs) migrate into the developing bone to establish their niche in the medullary cavity. Concurrently, bone-forming cells are distributed either along the inner surface of the bone, forming the endosteum, or on the outer surface of the bone, assembling the periosteum. These two mechanisms of bone formation also occur postnatally until the end of the growth period, as well as during the regenerative processes that follow a bone trauma [2,3,4].

Bone remodeling occurs during the entire lifespan through cycles of cellular activity that arise asynchronously throughout the skeleton. They lead to the zonal resorption of bone and to its replacement with newly formed bone. The bone remodeling process is vastly complex, but it is always a succession of the same steps—bone resorption by osteoclasts followed by bone matrix production by osteoblasts. Through this fundamental process, the skeleton constantly changes in response to mechanical stress and hormonal influences [5].

Following trauma, bone heals through the regeneration process, which is characterized by the formation of new bone [6]. Bone regeneration is a complex, well organized, and coordinated process. It ensures the recovery of bone shape and function without invading the scar connective tissue area [4]. Moreover, bone healing follows the same mechanism through which they were formed, including either intramembranous or endochondral ossification [7].

Bone repair involves the mobilization of adult skeletal stem cells, which are also termed progenitor cells. They synthesize the cartilaginous and/or bone material that is then deposited at the site of the defect. These cells originate from several sources. The most important are the periosteum, the endosteum, and the hematogenous marrow [8].

The periosteum envelops the outer surface of bones, and it is considered one of the most regenerative tissues for skeletal osteogenesis [9,10,11]. It supplies a vital environment for cortical bone growth, bone modeling, and fracture healing [12,13,14]. Regarding its structure, it is a thin layer of vascularized tissue that is substantially involved in maintaining bone strength, and supports the attachments of tendons and muscles [4]. The periosteum covers the entire surface of the bone, except for the articular surfaces and the insertion areas of ligaments and tendons. It is not present in sesamoid bones [15,16].

The periosteum is highly responsive to mechanical stress, and its preservation is crucial for normal bone repair [17,18]. It provides blood supply to bone tissue and has a paramount role in bone growth and repair [1]. Structurally, it is composed of two layers: (i) the external layer (that is, in turn, divided into a superficial layer with a well-vascularized and predominantly collagenous structure) and a deep layer with a fibro-elastic structure [12,19]; (ii) the internal layer (that is also named the osteogenetic layer) holds numerous osteoprogenitor cells, which are multipotent cells that generate osteoblasts through division and differentiation [19,20].

The thickness of the internal periosteal layer varies with age. It reaches its greatest thickness during fetal life, and progressively grows thinner during aging. As its thickness decreases, the vascularity also decreases, and consequently so does the number of perivascular pericytes [21]. This decrease in the number of osteoprogenitor cells and perivacular pericytes leads to a significant reduction in osteoinductive potential [12]. However, this osteoinductive potential never entirely disappears, and can be vividly reactivated in crisis situations such as fractures or surgically induced bone trauma [22].

Internally, the bone is lined by the endosteum, a periosteum-like membrane that covers all internal surfaces of bones, including the inner surface of the skull, bone trabeculae, Haversian systems, and Volkmann’s canals [19]. It consists of a layer of flattened osteoprogenitor cells and reticular fibers [5]. The endosteum is significantly thinner than the periosteum [23]. It is most developed in the fetus, where it is actively involved in bone growth, and becomes thinner and less active with age. It can be reactivated following bone injury, as well [1].

Hematogenous marrow is present in bone cavities, and it is composed of hematopoietic stem cells, stromal cells, endothelial cells, pericytes, fibroblasts, adipocytes, and numerous blood vessels [20,24].

It is known that cells that originate from these previously described three tissues generate different types of healing, i.e., either intramembranous or endochondral ossification. Thus, periosteal lesions heal by endochondral ossification, while bone marrow lesions heal by intramembranous ossification [8].

Certain studies have provided direct evidence that the periosteum, the endosteum, and the bone marrow are major sources of skeletal stem cells. However, they contribute differently to osteogenesis and chondrogenesis during bone repair [8,18,25,26,27]. Some reports indicate that the cells in the periosteum, the endosteum, and the bone marrow are not identical in their sensitivity to mechanical or biological stimuli [28,29]. The different cellular contributions of the periosteum, of the endosteum, and of the bone marrow to osteogenesis suggest intrinsic differences both in the resident stem cell populations and in their tissue microenvironment [8].

Considering all these aspects, the present study aims to assess whether the healing processes at the bone–implant interface begins at the periosteal level when the implant penetrates the hematogenous marrow. Besides this specific aspect that is investigated, the involved phenomena are much more complex, and they comprise a variety of factors, including those related for example to the impact of the implant surface characteristics on osseointegration, as surface properties are critical for bone–implant interface healing and may influence the outcomes of the process. However, by using the same types of (appropriate) implants, such variabilities may not influence the results of the present study.

Our working hypothesis was that the bone formation at the bone–implant interface begins at an endosteal level if the implant penetrates the bone marrow. This hypothesis was formulated based on the authors’ previous observations after long-term experiments on rabbits [30,31]. Thus, it has been observed that periosteal proliferation occurs in later stages. However, the precise onset of periosteal proliferation has not been determined so far, either by us or in the literature. Therefore, in the present study, our scope was to investigate this phenomenon and to extract rules-of-thumb for further clinical approaches.

## 2. Materials and Methods

This study was approved by the Bioethics Commission of the University of Agricultural Sciences and Veterinary Medicine in Cluj-Napoca, with Approval no. 384/20 August 2023. The requirements of the Declaration of Helsinki, the Protocol of Amsterdam, the Directive 86/609/EEC, and the Government Ordinance 37/2002 with reference to the protection of experimental animals were respected. The ARRIVE List is provided in the Supplemental Material.

The biological material was represented by 8 male cross-breed rabbits, which were 11 months old and raised in the Biobase of the Faculty of Veterinary Medicine of the University. This number of experimental animals was carefully determined by the Bioethics Commission in order to be able to achieve statistically relevant results, while avoiding unjustified animal sacrifice. Thus, the statistical analysis carried out before the study revealed that considering 8 rabbits would generate an effect size of 1.342 and a statistical power of 80–95%. The (purely) histological nature of the investigation was also highlighted in the analysis performed by the Bioethics Commission. Therefore, a cohort of eight subjects was considered sufficient for the present study.

The rabbits benefited from a temperature of 20 to 24 °C, natural light 12 h per day, as well as granulated food and fresh water at discretion.

**Figure 1:**
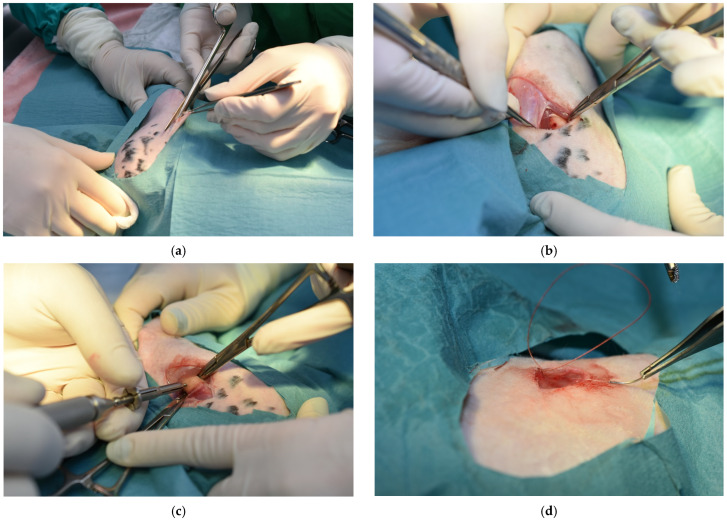
Phases of the surgical procedure followed to insert the titanium implant: (**a**) incision line and access created to the femural bone; (**b**) implant hole generated in the femural bone; (**c**) placement of the implant; (**d**) closure of the wound.

Custom-made titanium self-tapping screws (Biomicron Transilvania, Cluj-Napoca, Romania) with a length of 5 mm and a diameter of 2 mm were utilized for the surgical procedures. The main steps of these procedures are presented in Figure 1.

We correlated the size of the screws with the indications found in the literature, which recommends the following maximum parameters accepted for endosseous implants in rabbits: 6 mm in length and 2 mm in diameter. Implants used in larger species, such as humans, are not suitable for relatively small species. Using wider implants would exaggeratedly increase the load, leading to a higher risk of subsequent bone fracture [30]. Implants insertion was carried out in such a way as to penetrate both the periosteum and the endosteum, allowing us to assess the bone reparation processes in a comparative way. We consider that a group of subjects with mere periosteal penetration would not have provided additional information. In contrast, it would have only complicated the investigation, and it would also have increased the number of sacrificed animals.

Anesthesia pre-medication and induction were performed with an intramuscular injection of 5 mg/kg xylazine, and 35 mg/kg ketamine, followed by isoflurane mask maintenance. Fluid therapy was administered during the intervention at the maintenance rate. Following clipping and antisepsis of the surgical field, the skin and the subcutaneous tissue were incised, and the femoral surface was exposed. A hole was drilled through the distal third of the femoral diaphysis with a 1.8 mm diameter drill, and then the screw was inserted, followed by a routine closure. Enrofloxacin 20 mg/kg and meloxicam 1 mg/kg were administered by subcutaneous injections for 5 and 3 days, respectively.

Post-operative clinical examinations were performed daily until euthanasia. During the first two days, the rabbits preferred the decubital position most of the time. The animals did not refuse food and water, but movement towards the food and water containers was achieved with some difficulty in the first two days. This was due to a high-grade lameness, which began to diminish after the third day. This resulted in the animals becoming increasingly more active. Obvious wound-site swelling was noted during the first 24 h, but afterwards this gradually subsided in intensity. The animals were humanely euthanized 14 days post-operatively by sodium pentobarbital intravenous overdose, and the femur was extracted by disarticulation. The bone was incised 5 mm proximal and distal to the implant. The samples thus collected were immediately immersed in formaldehyde solution for histological fixation.

Histological processing was achieved by the fixation of each sample with 10% formaldehyde, decalcification with 7% trichloroacetic acid, dehydration with alcohol, clarification in 1-butanol, embedding in paraffin, and finally sectioning at 5 µm and Trichrome Goldner staining. To avoid decalcification artefacts, we utilized a slow decalcification method, which adequately preserves the bone structures.

An Olympus BX41 microscope (Evident Scientific, Tokio, Japan) equipped with an Olympus E-330 digital camera (Olympus Corporation, Shinjuku, Japan) was utilized for the examination of the histological preparations. Histological sections that intercepted the bone implant interface in the central area were selected to perform measurements. Serial sections were made, and 10 slides per animal were chosen, especially those that intercepted the working area in the most central possible position.

The evaluation of the microscopical slides was carried out by two experienced examiners who checked each other regarding performed observations.

All measurements were performed using the ToupView 4.11 software of the microscope, following the method described in [31]. Linear and polygonal tools were employed to determine the histological landmarks. Thus, the length of the osteal interface D_1_ (µm) and the length of the endosteal (medullary) interface of the implant D_2_ (µm) were measured. Further on, the surface areas of the proliferated bone in the osteal and endosteal (medullary) areas were determined, i.e., S_1_ (µm^2^) and S_2_ (µm^2^), respectively. All these parameters are shown on the obtained images in the next section of the study. In each of the two regions (i.e., 1 and 2), the surface area occupied by the areolas was determined (expressed as a percentage). Other morphological parameters for this type of study would not be relevant.

Differences between the percentages of newly proliferated bone in the osteal area and in the endosteal (medullary) area were analyzed using GraphPad Prism 8. Data were tested for normality, and a paired *t*-test was conducted, with the significance level set at α = 0.05 for percentage data only (% of areolar surface from S1 and % from S2, respectively). The measurement method is replicable, since it utilizes images that can be revised every time it is required. The statistical analysis was conducted using the Jamovi 2.7.2 version, as well as the G-power (Jamovi 2.7.2 version) module for the power analysis.

## 3. Results

**Figure 2:**
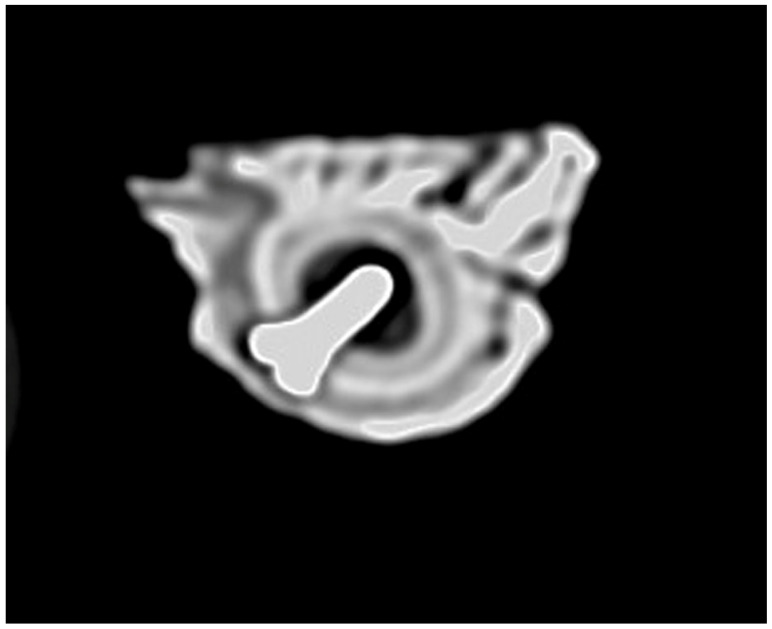
CT image of a titanium implant penetrating the medullary canal—example from one of the surgical procedures.

For all the surgical procedures performed, the CT scan revealed the correct positioning of the implant in the femoral bone, such that it penetrated the endosteum, entering the medullary canal for at least half of its length, as shown in the example in Figure 2.

**Figure 3:**
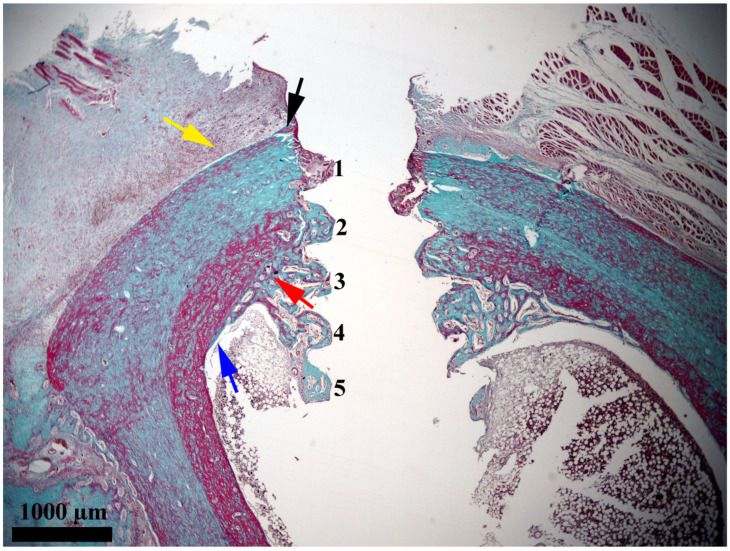
General feature of the bone–implant interface 14 days after the insertion of the implant. Notations: yellow arrow, periosteum; black arrow, newly proliferated bone in the periosteal area; blue arrow, endosteum; red arrow, newly proliferated bone in the endosteal area; 1, groove no. 1; 2, groove no. 2; 3, groove no. 3; 4, groove no. 4; 5, groove no. 5. Goldner’s trichrome stain was utilized.

After the insertion of the implants, a rapid evolution of bone proliferation processes at the bone–implant interface was observed at 14 days (Figure 3). At this point in the experiment, the newly proliferated bone was extended over most of the interface. It infiltrated into four of the six grooves between the turns of the implant, namely, grooves 2, 3, 4, and 5. Groove no. 1, which was the closest to the periosteum, was mostly occupied by augmentation material that consisted of bone fragments embedded in fibrin, which dated from the moment when the implants were inserted. Groove no. 6, which was located at the tip of the implant, was not yet filled with newly proliferated bone. Of the four grooves occupied by newly proliferated bone, three of them (i.e., no. 3, 4, and 5) were located in the medullary cavity, and one of them (i.e., no. 2) was located near the endosteum.

**Figure 4:**
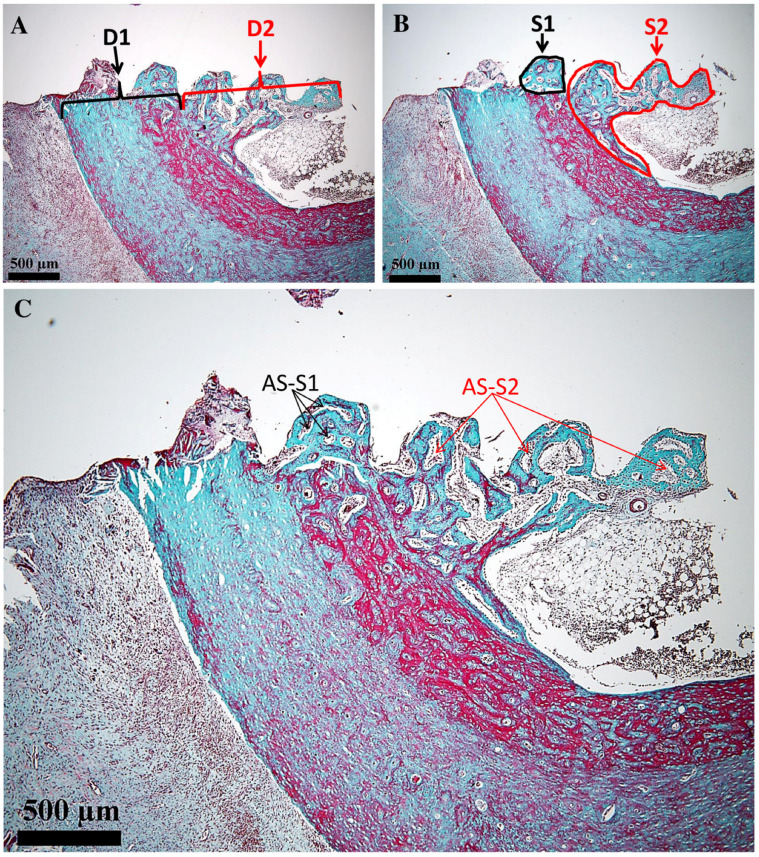
Histomorphometry of the bone implant interface. (**A**): D_1_ (µm)—black brace that represents the length of the osteal bone–implant interface; D_2_ (µm)—red brace that represents the length of the endosteal/medullary bone–implant interface. (**B**): S_1_ (µm^2^)—black freeform that represents the proliferated bone surface in the osteal area; S_2_ (µm^2^)—red freeform that represents the proliferated bone surface in the endosteal/medullary area. (**C**): AS-S_1_—black arrows that represent the surface (µm^2^) of the areolas in the proliferated bone of the osteal area; AS-S_2_—red arrows that represent the surface (µm^2^) of the areolas in the proliferated bone of the endosteal/medullary area. Goldner’s trichrome stain was utilized.

As pointed out in the previous section, we have considered a series of characteristic morphological parameters, as presented in Figure 4. D_1_ (µm) represents the length of the osteal bone–implant interface; D_2_ (µm) represents the length of the endosteal/medullary bone–implant interface; S_1_ (µm^2^) is the proliferated bone surface in the osteal area; S_2_ (µm^2^) is the proliferated bone surface in the endosteal/medullary area; AS-S_1_ (µm^2^) is the surface of the areolas in the proliferated bone of the osteal area; AS-S_2_ (µm^2^) is the surface of the areolas in the proliferated bone of the endosteal/medullary area.

The average length of the endosteal/medullary interface (D_2_, Figure 4) of the proliferated bone around the implant was approximately 25% longer compared to the osteal interface (D_1_, Figure 4), as presented in Table 1. The minimum length of the osteal interface (D_1_) was 1045 µm and the maximum length was 1363 µm. The minimum length of the endosteal/medullary interface (D_2_) was 1439 µm and the maximum length was 1614 µm (Table 1).

By applying the Student’s *t*-test for paired samples, the data shown in Table 2 were obtained. These data allowed for evaluating the statistical power of this study and for determining the number of subjects included in the study, using the J-power module of the Jamovi version 2.7.2. Moreover, the statistical power for eight rabbits, as considered for the study, with an effect size of 2.809 and α = 0.05, is 1 (Table 3).

The proliferated bone at the endosteal/medullary interface had a surface area almost 6 times larger compared to the one proliferated at the osteal interface of the implant (Table 4). The results of a paired samples *t*-test S_1_/S_2_ are presented in Table 5. One may observe that, for S_1_/S_2_ with an effect size of 7.011, α = 0.05, and eight subjects, the calculated statistical power equals 1.

The surface covered by the areolae (Table 6) in the two considered regions (S_1_ and S_2_) was as follows: in the osteal area (S_1_) the areolae covered 22.2% of the surface, while in the endosteal/medullary area (S_2_) the areolae covered 36.4% of the surface. A paired *t*-test has revealed a statistically significant difference between the areolar surface percentages of S_1_ and S_2_ (*p* < 0.0001), as presented in Table 6. The smallest percentage of areolar surface was 17.1% in S_1_ and 29.9% in S_2_. The highest percentage of areolar surface was 28.4% in S_1_ and 43.0% in S_2_.

The calculated effect size for the S_1_/S_2_ percent evaluation is shown in Table 7. For an effect size of 5365, α = 0.05 and eight subjects, the calculated statistical power equals 1.

## 4. Discussion

In the present study, after 14 days, a discrete activity of bone proliferation with a starting point at a distance from the interface and a clear tendency to extend towards it was observed in the periosteal area. The regenerated bone continued towards the interface and gradually narrowed. The portion in contact with the interface was thin, zonal, and consisted of bone in an early stage of proliferation (osteoid). This demonstrates a recent contact with the area. It should be noted that this segment had a zonal distribution; therefore, at that point in time it only covered a small part of the interface. Considering its appearance of young bone that has covered only small areas, it most likely represents the first bone to appear in the periosteal area of the interface. Therefore, we were able to conclude that when titanium implants are inserted in direct contact with the hematogenous marrow, the bone reparative processes with a periosteal starting point reach the interface with the titanium implant after approximately 14 days (Figure 4). This strategy of implant placing is recommended for bones rich in marrow, whereas in other cases, periosteal bone proliferation, although somewhat slower, should be relied on.

The interface area next to the unthreaded collar and next to the bone wall did not contain newly proliferated bone after 14 days. Significant bone proliferation could be seen beginning in the endosteal area, where the implant groove adjacent to the endosteum was filled with newly proliferated bone. In that area, the proliferated bone extended deep towards the medullary cavity, and it continued seamlessly around the intramedullary section of the implant.

In the implant grooves that exceeded bone thickness, protruding into the medullary cavity, the newly proliferated bone formed a truncated cone-shaped structure attached to the endosteum for a considerable distance. This structure was made up of trabecular bone, but the areolar size was different, and it depended on the distance from the endosteum. The half that was closest to the endosteum contained small areolae and thick trabeculae, while the deeper half had larger areolae and thinner trabeculae.

The proliferation of newly formed bone around the intramedullary section of the implant was, most likely, intense on account of the contact with the hematogenous marrow. This marrow provided abundant quantities of osteoblastic precursors and stimulating molecules. Moreover, this section of the implant was not located in the vicinity of affected bone. Instead, it was near the hematogenous bone marrow. Therefore, resources were not spent on replacing affected bone structures with newly proliferated ones. In contrast, the regeneration depended solely on the possibilities of bone proliferation that the marrow and the endosteum have been able to provide. In other words, bone proliferation near the implant section (that penetrates into the medullary cavity) benefited from superior conditions compared to the interface adjacent to the bone wall. In this rich microenvironment, bone proliferation was fast and intense. Thus, it resulted in a truncated cone-shaped bone structure that was organized around the implant, with its large base attached to the endosteum. In other words, the anchoring structure evolved faster than the coverage of the interface with newly proliferated bone. The presence of this bone structure to consolidate and anchor the implant at such a short time interval after its insertion provided some stability, with consideration of the moment of the investigation.

This structure was composed of young bone that was unable to provide the mechanical resistance necessary to support the implants. Despite this aspect, at this point in the experiment, this structure was the one that ensured the coating of the implant with newly formed bone on the largest surface of the interface. Also, it offered the best anchorage to the deep bone structures.

The stage reached by this bone structure after only 14 days post-insertion was, to a large extent, due to the fact that the implants were inserted with more than half of their length in direct contact with the hematogenous bone marrow. This fact was decisive both for the rapidity of formation and for the surprisingly large surface it came to cover. One can also consider it an adaptive response to the special situation created in the area, since the organism completed the cortical bone of the femur with a structure similar to medullary bone. This structure had not existed in the medullary cavity of the bone before this intervention.

It should be noted that all the newly proliferated bone had an endosteal and medullary starting point up to this point in the experiment. In contrast, in the periosteal area, the proliferation of new bone was still in its early stage.

Consequently, from a histological point of view, 14 days post-insertion was a favorable moment for the implant in terms of bone proliferation, but it was a moment of vulnerability regarding the resistance of the newly formed bone. As this bone was immature, it could not yet provide the necessary stability. The insertion of the implant in this way offered good primary stability that was ensured by the neighboring bone. At the same time, advantageous osseointegration conditions were provided by the direct contact with the hematogenous marrow. This contact also ensured the fast stimulation of bone reparative processes without the cells or stimulating factors encouraging alteration. Such alterations may depend on the utilized isolation procedure or on the harvested volume, as is the case when marrow aspirate or components extracted from the marrow are utilized [26,32].

Both models of osteogenesis have been seen in all areas of peri-implant bone healing. However, the speed of the process eminently favors contact osteogenesis, which is crucial for the osteointegration of dental implants. At the 14-day time point, the newly formed bone originated through contact osteogenesis. It extended along the implant surface, into the medullary cavity, where the implant was not surrounded by bone; therefore, distance osteogenesis could not have occurred.

The use of mesenchymal stem cells (MSCs) in regenerative medicine has attracted considerable attention due to their potential to restore tissues and organs to a normal, healthy state after injury or damage. This field of research has sought methods for stimulating the body’s intrinsic ability to heal itself, and to restore the original function of severely damaged tissue or organs. The stimulation of reparative processes can be achieved by several procedures: (i) by delivering stem cells that have been prelevated from a donor and multiplied in culture (the exogenous route); (ii) by mobilizing and recruiting stem cells from within the patient’s body (the autologous route); (iii) by transplanting organs grown in the laboratory from stem cells (tissue engineering) [33]. Bone marrow, adipose tissue, and the stromal vascular fraction of adipose tissue have been utilized as sources for harvesting such cells [34]. Despite the multiple advantages offered by the therapy with MSCs, there are some limitations: (a) cells derived from the hematogenous marrow and multiplied in culture have limited cellular activity in geriatric patients; (b) cells harvested from adipose tissue are immunogenic and genetically unstable; (c) cells harvested from the stromal vascular fraction of adipose tissue are heterogeneous and generate unreliable results [34].

MSCs have been originally identified as progenitors of skeletal tissues in mammalian bone marrow. Because of their differentiation capacity, these cells have been called osteogenic stem cells or bone marrow stromal stem cells [20]. They are also called skeletal stem cells (SSCs) because they are postnatal multipotent progenitors of skeletal tissues that are capable of generating the most important non-hematopoietic cell types associated with bone (e.g., osteoblasts, chondrocytes, marrow adipocytes, fibroblasts, and stromal cells from the bone marrow) [35,36].

The name mesenchymal stem cells was assigned by Caplan in 1991 [37]. He included here a class of cells that exist in the bone marrow and periosteum in humans and in other mammals. Such cells can be isolated and multiplied in culture, and they maintain in vitro their ability to form a variety of phenotypes and mesodermal tissues [38]. Multiple researchers have embraced bone marrow stem cell therapy, in the hope that they can cure diseases by differentiating into local cell types [39,40]. Similar cells have also been isolated from other tissues such as the umbilical vein, saphenous vein, bones, skeletal muscles, lungs, dental pulp, adipose tissue, and placenta [20].

Other cells that are considered to have a remarkable potential to support reparative processes are perivascular pericytes. They are undifferentiated connective tissue cells, and were described in 1873 as a population of contractile cells associated with the walls of small blood vessels [20,41]. Due to the fact that blood vessels are present in almost all organs, pericytes can be obtained from several sources. Because they possess stem cell-like qualities, pericytes are considered by some authors to be their equivalent [42], or perhaps their precursors [43]. Pericytes regulate blood flow and play a decisive role in angiogenesis and vascular maturation, as well as in remodeling and permeability. Also, pericytes collaborate with astrocytes to maintain the functional integrity of the blood–brain barrier [44]. Pericytes have a relationship with endothelial cells, but also with tissue-specific stem cells, constituting stem cell niches [38]. In addition, pericytes have been shown to play a role in the niche maintenance of hematopoietic stem cells in the bone marrow [45].

Because there is currently a lack of specific markers to clearly differentiate pericytes from stem cells, it is difficult to say whether these cells represent in vitro and in vivo counterparts of the same cell population. What can be stated with certainty is that pericytes contribute to angiogenesis in early repair processes [33].

Some researchers have found that there are multiple populations of MSCs that have biological properties that are affected by their tissue of origin [46]. Furthermore, during culture, MSCs undergo changes. Therefore, there is no certainty that after the culture the cells will remain identical to those found in vivo [20,47]. Some authors state that MSCs are a highly heterogeneous mixture of cells of which only a fraction has differentiation potential. They further point out that these cells have been tested to treat a wide range of conditions, often in unscientific, exploitative, and potentially dangerous ways [48,49].

Two decades after introducing the term MSC, Caplan proposed to change it because of its frequent misinterpretation. He argued that the term was often interpreted as if MSCs are guaranteed to bring medical benefits and to differentiate into cells capable of the specific repair of the damaged tissue. This interpretation has induced the misplaced hope that a stem cell treatment may cure severe medical conditions, which range from osteoarthritic knees to neurological conditions that include dementia [38]. These misinterpretations have led some practitioners to oversell stem cell treatments, claiming that they can cure the blind, make the lame walk, and reverse the aging of old tissue [50].

Thus, Caplan proposed to change the name to medicinal signaling cells to indicate that these cells secrete bioactive factors upon reaching the affected area. This entails an immunomodulatory or trophic action. Specifically, MSCs that arrive at the injured area rarely or never differentiate into site-specific cells [51]. Instead, they secrete bioactive factors with important therapeutic effects [35]. Based on these reasonings, many authors have argued that it is the patient’s own site- and tissue-specific resident stem cells that form the new tissue, after significant stimulation by bioactive factors secreted by exogenously supplied stem cells [27,52]. We agree with this point of view, and consider that the marrow cells support the reparative processes in the immediate vicinity, as was the case in the present study. These cells do not undergo changes because they are not manipulated or subjected to operations that affect their integrity or functionality.

Fractures are the most common bone trauma, most of which heal with appropriate treatment. However, approximately 5 to 10% do not heal [53]. Treatment methods such as bone grafting, the delivery of growth factors, and cell-based therapies are utilized to stimulate fracture healing [54]. The use of stem cells in fracture repair has been extensively studied. Although they are of endogenous origin, MSCs are a heterogeneous population that is present in several tissues, including in the periosteum. Their positive effect in fracture repair is known, but whether MSCs that originate from different sources have the same functional properties is a question that has sparked much controversy [54].

The periosteum and the bone marrow are highly involved in the fracture repair process, and some authors claim that the periosteum is the more important structure. They argue that fractures can heal in the absence of bone marrow, whereas the removal of periosteum would cause non-union [54]. This point of view is also shared by other authors, who claim that the main cellular contributors to callus formation are periosteal cells [55]. Moreover, it has been reported that periosteal cells participate in both endochondral and intramembranous ossification, while medullary cells solely participate in intramembranous ossification [8]. The periosteum contains cells that are responsible for the growth, shaping, remodeling, and healing of bone fractures [21]. According to some authors, the periosteum contains SSCs with an increased capacity for cell growth and clonogenicity, as well as with superior regenerative capacities compared to BMSCs [4].

Since most of the callus tissue is arranged subperiosteally, it is not a surprise that most of the regeneration cells originate from the periosteum. A study concluded that in callus fracture repair processes, bone regeneration is highly dependent on the periosteum, and to a lesser extent on the bone marrow [56]. It is estimated that 70% of the regenerated cell population in callus-healed fractures originate from the periosteum [57]. Numerous clinical and animal model studies have shown the special osteogenic capacity of the mature periosteum. Periosteal grafts have been successfully utilized in the treatment of pseudarthrosis, infected bone lesions, large bone defects, and non-union fractures [58,59].

By following the outcomes for cells that are derived from the periosteum, bone marrow, and endosteum, a study has concluded that skeletal progenitor cells are recruited locally and concurrently from these three tissues. The author explained that the periosteum, the bone marrow, and the endosteum generate osteoblasts, while chondrocytes are formed solely on the account of the periosteum. This study concluded that the periosteum, the endosteum, and the bone marrow contain pools of progenitor cells with distinct osteogenic and chondrogenic potentials, which vary depending on the tissue environment. Also, it pointed out that cells that participate in bone repair processes are mostly recruited locally [8].

After excluding the possibility that the cells that are involved in bone reparative processes are brought through the circulatory system, it was concluded that they are of local origin. Additionally, periosteum-derived cells were always found on the periosteal surface and bone marrow-derived cells were always found in the medullary cavity, while the participation of cortical bone was limited [56]. Cartilaginous cells that participate in callus formation are almost always derived from the periosteum, whereas osteoblasts are derived from both the periosteum and the bone marrow [60]. Some authors point out that separating the role of different cell sources during skeletal regeneration is often difficult due to the complexity of the structures. In most cases of bone injury, the cortical bone is interrupted and allows communication between the periosteum, endosteum, bone marrow, and surrounding soft tissues. Therefore, the healing response in one of the tissues can affect the response in the adjacent tissue through the diffusion of cells and growth factors [8].

The endosteum is a potent source of osteoprogenitor cells, and under certain conditions it may be a more potent source than the periosteum [61,62]. This is the case with metaphyseal or epiphyseal fractures that involve cancellous bone, where the highly vascularized environment promotes faster fracture healing compared to compact bone fractures [30]. Some authors claim that endosteal cells along with BMSCs account for more than 60% of the newly proliferated bone [31].

As illustrated above, most authors agree that the periosteum, endosteum, and bone marrow participate in bone reparative processes [63,64]. However, opinions regarding their levels of participation differ. The only explanation that may account for these differences is that the repair processes have been analyzed under different conditions. The reparative processes that occur in fractures differ from those that occur in zonal bone defects associated with accidents, and even more so from those caused experimentally.

In the present study, a titanium implant was inserted into a hole created without a major tissue loss. Thus, it resulted in an intimate contact between the implant and the bone wall. Additionally, the physical barrier between the periosteum and the endosteum bone marrow prevented the transfer of cells between them. Hence, the potential of the endosteum and of the bone marrow to participate in the reparative processes of the periosteum could be assessed individually.

It was observed that 14 days after the insertion of the implants in direct contact with the hematogenous marrow, the reparative processes were present in 55.7% of the interface. The chosen moment facilitated a precise evaluation of the unique participation of the periosteum, the endosteum, and the bone marrow in the proliferation of new bone at the bone–implant interface. Since the reparative processes did not yet cover the entire interface between the new bone of periosteal origin and the bone of endosteal-medullary origin, there was a segment of untouched interface. The present study found that 85.61% of the newly proliferated bone was of the endosteal-medullary area, while that of the osteal area covered confined areas that did not reach 15%. Therefore, we conclude that in early osseointegration, the proliferation of bone of endosteal-medullary origin is predominant, while the proliferation of that of periosteal origin is merely incipient.

In consequence, we may advise that when the bone in which an implant is to be inserted contains hematogenous marrow, the implant should be lengthy enough to penetrate the marrow.

Based on the existing literature, the time required for bone to achieve sufficient maturation in order to be able to sustain its functional loading varies between three and six months. This time interval depends on numerous factors such as bone quality and sex. The latter impacts osseointegration, as the necessary time is shorter for males than females.

At the 14-day mark, it is hazardous to establish the optimal healing time, i.e., the time interval that would allow for safe implant loading. Although this strategy of inserting the implant assures a significant increase in the bone implant interface, the newly proliferated bone requires time in order to follow the sequential phases—osteoid to primary bone (bone tissue) and remodeling to the formation of lamellar bone, which is the only form that provides the mechanical resistance that is necessary to support prosthetic rehabilitation.

The immediate loading protocol recommendations follow the same principle, namely, sufficiently long implants must be utilized in order to penetrate the bone marrow in bone marrow-rich areas.

The present study did not aim to monitor the inserted implants in their dynamics in contact with the bone marrow. The aim has been to observe the onset of endosteal-medullary and periosteal reparation processes, as well as the situation after 14 days. We tried to report these aspects, and we refrained from making assumptions regarding long-term models. Thus, we cannot claim that bone marrow penetration is necessary for implant stability, nor for primary stability. We only affirmed that contact with the hematogenous bone marrow can influence the onset of the osteointegration process, significantly increasing the bone implant interface.

It is not the scope of this study to deny the importance of the periosteum in bone reparative processes, but merely to emphasize the fact that the endosteum plays a greater role in the bone proliferation process, along with the hematogenous marrow. However, the importance of the periosteum is all the greater when the area to be repaired contains little or no marrow. In such a scenario, the periosteum would become the main source of cells that participate in the reparative process. However, the reparative processes would be slower without the participation of endosteum and hematogenous marrow.

We may conclude that our working hypothesis was confirmed. Thus, the bone formation initiates at the medullary endosteal region when the implant penetrates the hematogenous bone marrow. Furthermore, addressing interspecies differences requires comparative studies, which must consider the existing variations between small-scale animals (i.e., mice or rats) and larger-scale animals (such as sheep, goats, or pigs) regarding the structural particularities of their bones.

A limitation of this study seemed to be the small number of animals (i.e., rabbits) authorized for investigation by the ethics committee. However, even in such a situation, one must point out that a larger number of rabbits would have improved only the statistics of the results, and not the obtained histological aspects.

As determined in the statistical analysis, the effect size necessary for a paired *t*-test is 1.342, considering eight subjects, a statistical power over 0.9 (i.e., a power of detection of 80–95%, equal to the probability of detecting), and a type 1 error rate of 0.05. An effect size larger than 1.496 would lead to the statistical power of the study surpassing 95% (i.e., almost certain detection). In our study, the calculated ES for D_1_/D_2_ is 2.809, for S_1_/S_2_ it is 7.011, and for %S_1_/S_2_ it is 5.365. This corresponds to a statistical of over 0.9. From our analysis, we obtained an estimated statistical power of 80–95%. However, the statistical power obtained after computing our data is higher than 95%.

This does not necessarily mean that future work must use a larger number of animals in different physiological states. It is not expected that such an approach would generate more conclusive results. Rather, experiments on larger animal species that allow for implant insertion into their maxillary bones could bring about more valuable data.

Regarding the clinical application of the conclusions, although the results obtained in animal studies cannot be fully extrapolated to humans, the obtained data can be useful. Therefore, our conclusions are merely recommendations for clinical activities. Further studies can validate our conclusions in clinical practice, even in the absence of histological confirmation. The extension of the newly formed bone on the surface of the implant in the medullary cavity with the significant increase in interface can also be assessed using imaging techniques such as CT or optical coherence tomography (OCT) [65,66]. Such approaches are also the subject of future work in our groups.

## 5. Conclusions

In the present study, when an implant was inserted in contact with the hematogenous marrow, nearly all the newly proliferated bone at 14 days post-implantation had an endosteal starting point and was under the direct stimulation of the hematogenous marrow, while at the periosteal interface, the proliferation of new bone was only incipient. Furthermore, in the area of contact between the implant and the hematogenous marrow, bone proliferation was facilitated and benefited from superior conditions compared to the bone wall and periostal interfaces. Based on the results of this experiment, we are tempted to conclude that the hematogenous marrow that exists in the medullary spaces supports the early bone proliferation processes around the implants at a level that, in our opinion, is difficult (or probably impossible) to reach with any other materials that may be utilized for this purpose.

Following the histological analyses, we conclude that the bone tissue that proliferates on the endosteal and marrow surface of the implant significantly increases the bone–implant interface and creates the setting for a good secondary stability. These findings suggested several clinical implications, including the fact that penetrating the bone marrow during the insertion of short implants results in a significant increase in the bone–implant interface, while the flap should be carefully managed. Also, the integrity of the periosteum must be maintained, as this is a key anatomical structure in cases of deficient bone marrow (i.e., severe mandible atrophy or vertical ridge augmentations).

A deep understanding of the osseointegration process is useful for the clinician in choosing the right implant in terms of length and positioning. 

## Figures and Tables

**Table 1:** Histomorphometry of the length of the bone–implant interface.

	D_1_ (µm)	D_2_ (µm)
Minimum	1045	1439
Maximum	1363	1614
Range	317.9	175.8
Mean	1219	1533
Standard Deviation	93.71	79.14
Standard Error of Mean	33.13	27.98
Coefficient of Variation (%)	7.69	5.16

**Table 2:** Paired samples *t*-test for the D_1_/D_2_ comparison.

								95% Confidence Interval				95% Confidence Interval	
			Statistic	df	*p*	Mean Difference	SE Difference	Lower	Upper		Effect Size	Lower	Upper
D_2_ (µm)	D_1_ (µm)	*t*-test	7.946	7.0	<0.001	314.522	39.581	220.928	408.116	Cohen’s d	2.809	1.199	4.391

**Table 3:** Statistical power analysis for D_1_/D_2_.

A Priori Power Analysis			
	User Defined		
Power	N	Effect Size	α
1.000	8	2.809	0.050

**Table 4:** Histomorphometry of the surface of the bone proliferated at the bone–implant interface.

	S_1_ (µm^2^)	S_2_ (µm^2^)	Areolar Surface S_1_ (µm^2^)	Areolar Surface S_2_ (µm^2^)
Minimum	91,639	501,311	16,307	147,156
Maximum	108,292	676,762	30,484	282,103
Range	16,652	175,451	14,177	134,947
Mean	101,297	602,348	22,639	222,158
Standard Deviation	6241	77,433	5063	54,440
Standard Error of Mean	2207	27,377	1790	19,247
Coefficient of Variation (%)	6.16	12.86	22.36	24.50

**Table 5:** Paired samples *t*-test for S_2_/S_1_.

			Statistic	df	*p*	Mean Difference	SE Difference		Effect Size
S_2_ (µm^2^)	S_1_ (µm^2^)	Student’s *t*	19.830	7.000	<0.001	501,051.483	25,267.898	Cohen’s d	7.011

**Table 6:** Distribution of areolar surface areas of osteal (S_1_) and endosteal/medullary (S_2_).

	S_1_ Areolar (% from S_1_)	S_2_ Areolar (% from S_2_)
Minimum value	17.1	29.4
25% percentile	18.13	31.28
Median value	22.5	37.35
75% percentile	24.85	40.5
Maximum value	28.4	43
Mean	22.2	36.4
Standard Deviation	3.90	4.94
Standard Error of Mean	1.38	1.75
Lower 95% CI	18.94	32.27
Upper 95% CI	25.46	40.53
Paired samples *t*-test, *p* value S_2_ areolar % from S_2_ vs. S_1_ areolar % from S_1_	<0.0001	
*p* value summary		
Significantly different (*p* < 0.05)?	Yes	
One- or two-tailed *p* value?	Two-tailed	
t, df	t = 6.383, df = 14	

**Table 7:** Paired samples *t*-test.

Student’s *t*-Test							95% Confidence Interval			
		Statistic	df	*p*	Mean Difference	SE Difference	Lower	Upper		Effect Size
S_2_ areolar % from S_2_	S_1_ areolar % from S_1_	15.175	7.000	<0.001	14.200	0.936	11.988	16.413	Cohen’s d	5.365

## Data Availability

The raw data generated throughout this study can be obtained from the first author.

## Supplementary Materials

The following supporting information can be downloaded at: https://www.mdpi.com/article/10.3390/medicina61101841/s1, ARRIVE List [67].
